# Towards a balanced view of the bacterial tree of life

**DOI:** 10.1186/s40168-017-0360-9

**Published:** 2017-10-17

**Authors:** Frederik Schulz, Emiley A. Eloe-Fadrosh, Robert M. Bowers, Jessica Jarett, Torben Nielsen, Natalia N. Ivanova, Nikos C. Kyrpides, Tanja Woyke

**Affiliations:** Department of Energy Joint Genome Institute, Walnut Creek, California USA

**Keywords:** Bacteria, Bacterial diversity, Tree of life, Small subunit (SSU) rRNA, Candidate Phyla Radiation (CPR), Microbial dark matter (MDM), Metagenomics, Novel bacterial lineages

## Abstract

The bacterial tree of life has recently undergone significant expansion, chiefly from candidate phyla retrieved through genome-resolved metagenomics. Bypassing the need for genome availability, we present a snapshot of bacterial phylogenetic diversity based on the recovery of high-quality SSU rRNA gene sequences extracted from nearly 7000 metagenomes and all available reference genomes. We illuminate taxonomic richness within established bacterial phyla together with environmental distribution patterns, providing a revised framework for future phylogeny-driven sequencing efforts.

**Electronic supplementary material:**

The online version of this article (10.1186/s40168-017-0360-9) contains supplementary material, which is available to authorized users.

## Main text

Bacteria are major drivers of global biogeochemical cycles, impacting the environment, animal and plant health, and the evolutionary trajectory of life on Earth. Modern molecular approaches have provided a means to construct an increasingly detailed catalog of global bacterial diversity. In particular, the small subunit ribosomal ribonucleic acid (SSU rRNA) gene has for a long time been considered the gold standard for molecular taxonomic classification of bacteria and is still widely being used [1, 2] despite some limitations [3, 4]. Amplicon-independent approaches such as genome-resolved metagenomics and single-cell sequencing allowed researchers to overcome some of the previous barriers and promoted the discovery of unknown bacterial lineages at an unprecedented pace [5–7], seemingly reaching a climax with currently more than 140 proposed bacterial phyla [8, 9]. However, much bacterial diversity is still largely unaccounted for due to limited availability of genome data and inherent biases and chimeras in amplicon data.

**Fig. 1:**
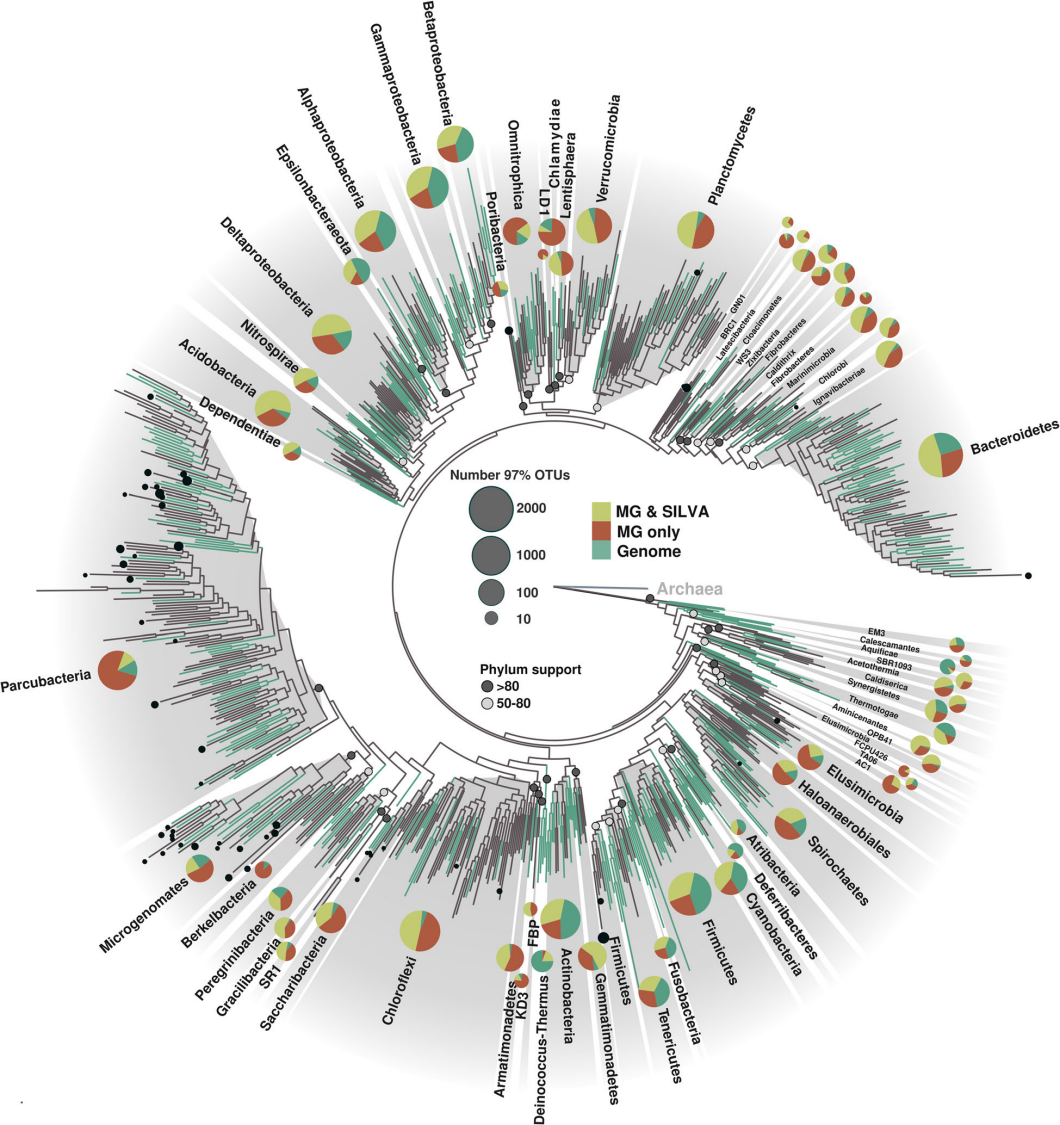
Bacterial SSU rRNA gene-based maximum-likelihood phylogenetic tree. Shown are representative taxa based on all SSU rRNA genes ≥ 1200 bp extracted from 6744 metagenomes deposited in IMG/M [10] and reference genomes (isolates, single amplified genomes, and metagenome-assembled genomes), which were consecutively de-replicated at 97% (97% OTUs) and then clustered at 85% sequence similarity thresholds (85% clusters). Bacterial phyla represented by at least two 85% clusters are shown, and for each phylum, bootstrap support is indicated if greater than 50. Branches in the tree are colored in turquoise if clusters contain SSU rRNA genes extracted from genome sequences. Sizes of pie charts correlate with the total number of 97% OTUs in the respective phylum (log2 transformed) and are divided based on percentage of 97% OTUs assigned to any of the three categories: (1) OTUs consisting of metagenomic SSU rRNA sequences with at least one member found in the SILVA database (MG & SILVA, yellow), (2) OTUs consisting solely of metagenomic sequences derived from this study (MG only, red), and (3) OTUs containing SSU rRNA genes extracted from reference genomes. Eighty-five percent clusters marked with filled black circles would likely have been missed by amplicon studies using the Earth Microbiome primer set (515f/806r) [14], with the size of the filled circles correlating with the mismatch score (indicated are mismatch scores greater than 1 [4]). The tree was rooted based on a representative set of 15 archaeal SSU rRNA gene sequences

Building on the wealth of existing metagenomic sequence data, we depict a comprehensive and balanced view of the bacterial tree of life, a view neither affected by PCR-introduced artifacts and biases challenging amplicon data [2, 4] nor by overrepresentation of certain clades [7, 8], as achieved by data de-replication after clustering. We constructed a robust phylogeny from all SSU rRNA gene sequences extracted from metagenomes and high-quality reference genomes available through the Integrated Microbial Genomes with Microbiome Samples (IMG/M) system [10] (Fig. 1, Additional file 1: Table S1). This collection of about 124,000 bacterial sequences (~ 64,000 from metagenomes and ~ 60,000 from reference genomes) with a length of at least 1200 bp was first de-replicated at 97% nucleotide identity, followed by clustering at 85% identity that approximates order level lineages [11]. To assess novelty of our metagenomic SSU rRNA sequences, both 97% operational taxonomic units (OTUs) and 85% clusters were matched against the comprehensive SSU rRNA database SILVA [11]. In total, 11,278 97% OTUs were of sole metagenomic origin (“MG-only”), of which 4166 were completely novel with no match in the SILVA database [12] and 2826 97% OTUs had at least one cluster member derived from a genome sequence. To avoid the inflation of taxonomic richness and phylogenetic diversity (PD) by chimeric SSU rRNA sequences, which can range up to 70% in certain taxonomic groups [3] and 13% of SILVA-only 85% clusters (Additional file 2: Figure S1), SILVA-only OTUs (37,066 97% OTUs and 1266 85% clusters) (Additional file 3: Figure S2) were excluded from the analysis and tree building. Despite the low phylogenetic signal of the SSU rRNA gene as compared to concatenated alignments of marker proteins [13], the phylogenetic tree constructed based on the genomic and metagenomic SSU rRNA genes resolves the majority of established bacterial phyla (Fig. 1). Importantly, its topology roughly recapitulates phylogenies from concatenated alignments of single copy marker proteins [8, 9, 13].

Our analysis depicts that the most exhaustively sampled bacterial lineages are Alpha-, Beta- and Gammaproteobacteria and Epsilonbacteraeota, both in terms of PCR-based surveys and genomic representation. Most of the 85% clusters contain at least one SSU rRNA gene extracted from a reference genome (Fig. 1), and metagenomic SSU rRNA sequences only marginally extend PD of these lineages (Additional file 4: Table S2). In addition, more than three quarters of 97% OTUs in these proteobacterial classes contain SSU rRNA genes detected in PCR-based studies and appear in the SILVA database [12]. Other well-sampled bacterial phyla include the Cyanobacteria, Bacteroidetes, Firmicutes, Actinobacteria, Aquificae, Deinococcus-Thermus, Thermotoga, Synergistetes, Atribacteria, Deferribacteres, and Fusobacteria with genome sequences available for the majority of order-level lineages and more than a third of all 97% OTUs (Fig. 1).

Our newly added metagenome-derived SSU rRNA gene sequences greatly expand the bacterial tree of life, making up one third of all 97% OTUs (Fig. 1, Additional file 4: Table S2) and increasing the PD of bacterial phyla by an average 70% (Additional file 4: Table S2). It becomes evident that large proportions of phyla with the greatest number of 97% OTUs, in particular Parcubacteria, Chloroflexi, Planctomycetes, Verrucomicrobia, and Acidobacteria, exclusively encompass our novel metagenomic SSU rRNA gene sequences, with up to 75% in the Parcubacteria (Fig. 1, Additional file 4: Table S2) and an increase in PD of more than 300% (Additional file 4: Table S2). Other bacterial phyla comprising a large proportion of novel sequences solely from this study include Chlamydiae, Omnitrophica, Elusimicrobia, Saccharibacteria, Armatimonadetes, and Microgenomates, all of which had more than 50% of their taxonomic breadth hidden in metagenomes. In particular, within the Parcubacteria, Microgenomates, and Saccharibacteria, many of the novel metagenomic lineages would not be amplified with commonly used universal primers [14] due to mismatches, as illustrated by their primer binding mismatch scores (Fig. 1). In addition, many SSU rRNA gene sequences of members of these phyla contain long insertion sequences [7]. The underrepresentation of these so-called “blind spots” in PCR-based studies is thus not surprising [4, 7]. Importantly, many of the sequences in clusters without associated reference genomes are readily available in metagenomic data for further exploration and potentially double total genome representation of order-level branches in the bacterial tree of life.

Beyond the expansion of the bacterial tree by novel metagenomic SSU rRNA gene sequences, our survey reveals the relative contributions of established phyla to the overall taxonomic richness within the domain bacteria (Fig. 1, Additional file 4: Table S2). Based purely on the number of the 97% OTUs, Bacteroidetes accounts for the phylum with the greatest taxonomic richness (1892 97% OTUs), followed by Firmicutes (1507 97% OTUs), Gammaproteobacteria (1269 97% OTUs), Alphaproteobacteria (1113 97% OTUs), and Chloroflexi (960 97% OTUs). However, when considering the 85% order-level clusters, as reflected in the number of branches in our phylogenetic tree and less affected from oversampling of certain bacterial groups, Parcubacteria is the major contributor to the bacterial tree (148 85% clusters), trailed by Bacteroidetes (81 85% clusters) and Chloroflexi (62 85% clusters). Parcubacteria is part of the originally proposed Patescibacteria [6], subsequently referred to as Candidate Phyla Radiation (CPR) [7] and recently proposed to exceed the combined diversity of all other bacteria [9]. Importantly, our SSU rRNA gene-based phylogeny, which is not limited by availability of genome sequences, suggests that the Patescibacteria/CPR does not make up more than 25% of branches (85% clusters) in the bacterial tree, even taking into account that the newly discovered metagenomic lineages from this study contribute to nearly 40% of its total size.

**Fig. 2:**
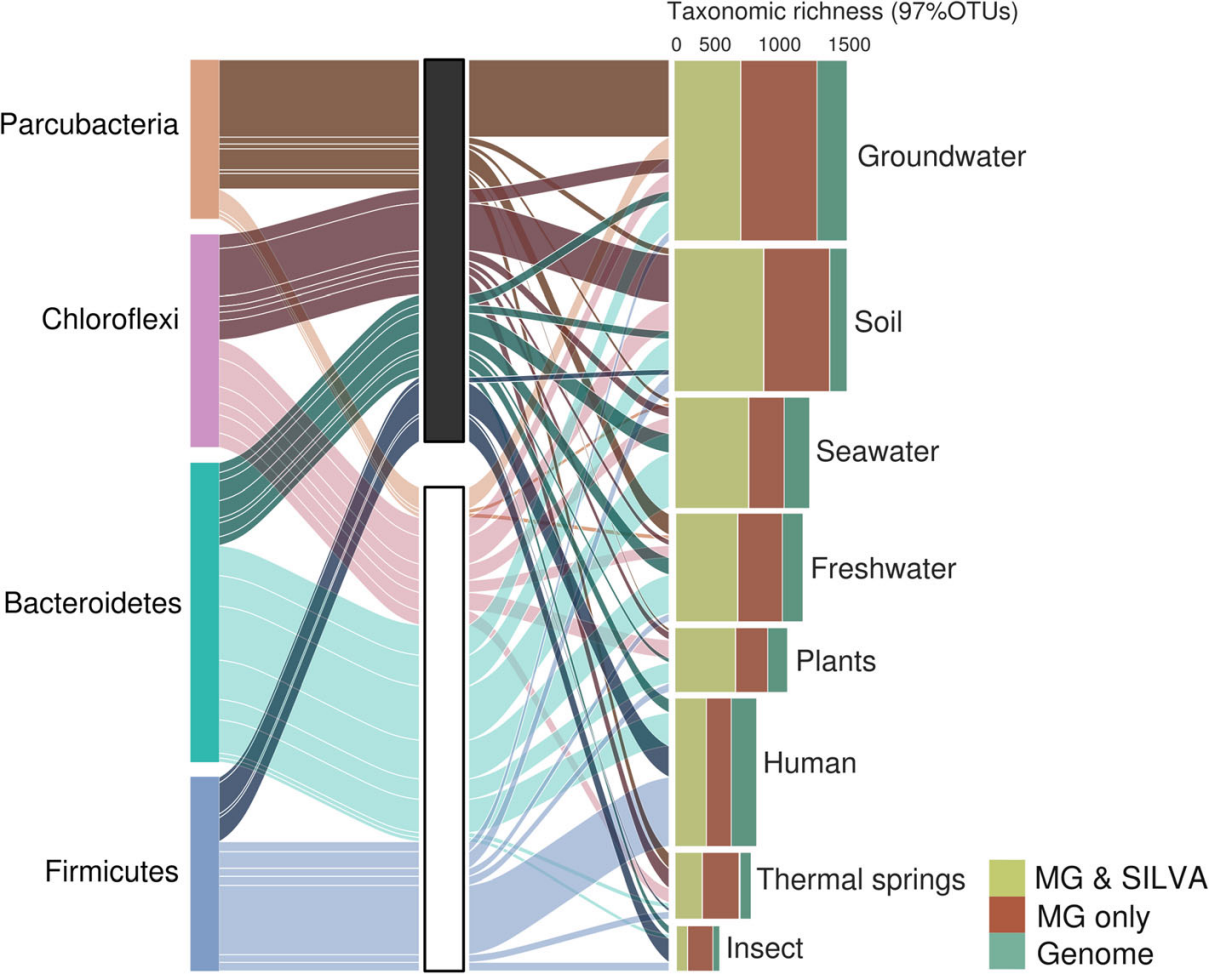
Environmental distribution of the four bacterial phyla with the highest taxonomic richness. For each phylum (left column), the proportion of all 97% OTUs derived from metagenomes exclusively (middle column, black) or observed previously either in the SILVA database or by genome sequencing (middle column, white) is indicated together with their environmental origin (right column). In instances where SSU rRNA gene sequences in 97% OTUs originated from different environmental categories, all categories were considered with a maximum count of one per environment. Connections between columns are only shown if representing more than ten 97% OTUs. Stacked bars illustrate total number of 97% OTUs in the respective habitat and either containing SSU rRNA gene sequences which include SILVA representatives (green), sequences derived from metagenomes exclusively (red), or from reference genomes (turquoise). We excluded the environment type “engineered” from this figure due to the heterogeneous origins of these samples

Associated metadata for all metagenomes available from the GOLD database [15] revealed environmental distribution patterns of all taxa (Additional file 1: Table S1). Samples surveyed with shotgun metagenomics might differ from sites typically targeted using amplicon data, and the degree of sampling effort likely impact observed taxonomic richness and PD. In metagenomic data, most taxonomic richness (97% OTU level) can be found in groundwater and soil (Fig. 2, Additional file 2: Figure S1). Parcubacteria are a major contributor in groundwater, Chloroflexi in soil, Bacteroidetes in fresh- and seawater, and Firmicutes in humans. While representatives of these phyla have been previously observed in these particular environments [6, 7, 9], our data suggests that most Parcubacteria occurring in freshwater and thermal springs have been unaccounted for thus far (Fig. 2). Other intriguing examples include the Chlamydiae and the Firmicutes. Our results illustrate that environmental reservoirs for novel Chlamydiae are mainly plants, soil, and freshwater, complementing an earlier study which showed that many new lineages are hidden in marine environments [16]. In the case of the Firmicutes, despite being extensively sampled and most human-associated members characterized, the majority of insect-associated members of this phylum was identified from the metagenomic data suggesting the presence of many novel lineages hidden in these hosts.

Our results shed light on current blind spots and underexplored branches in the bacterial tree of life. Novel lineages detected in this study likely bear unique metabolic capabilities and play crucial ecological and functional roles in their respective ecosystems. Improved cultivation-independent methods will be essential in conjunction with cultivation and functional characterization of amenable clades to elucidate their biology. By highlighting bacterial clades with scarce genomic information and associating their environmental origin, our study pinpoints environments that warrant additional sampling for targeted metagenome-resolved genomics, single-cell genomics and phylogeny-driven sequencing efforts.

## Methods

### Extraction, alignment, filtering, and clustering of bacterial SSU rRNAs

To extract SSU rRNA genes from all 6744 metagenomes (Additional file 1: Table S1) and from bacterial isolate genomes, single amplified genomes (SAGs) and metagenome-assembled genomes (MAGs) deposited in IMG/M [10], cmsearch from the Infernal package [17] was employed using the covariance model for the bacterial SSU rRNA molecule (RF00177) [18]. To ensure quality of SSU rRNA sequences and enhancing both aligned fraction for sequence clustering and increased phylogenetic signal for tree building, we removed sequences shorter than 1200 bp. The resulting 64,262 SSU rRNA sequences with a length of at least 1200 bp were aligned with cmalign [17] guided by the same SSU rRNA covariance model, using the --matchonly option, resulting in total alignment length of 1534 bp. Insertion sequences present in the SSU rRNA gene were removed during this process. Sequences with more than 40 gaps in the internal 900 bp present in all sequences in the alignment were removed resulting in a final set of 56,875 SSU rRNA sequences extracted from metagenomes and 56,461 from genomes available in IMG [10].

SSU rRNA gene sequences were clustered at a 97% sequence similarity threshold with usearch (version 9.1.13, -sort length -cluster_fast) [19] taking into account only the internal 900 bp (comprising V3-V7 variable regions) to avoid clustering artifacts due to terminal gaps. In the following, all 97% OTUs were clustered at an 85% sequence similarity threshold with usearch (version 9.1.13, -sort length -cluster_fast) [19]. Eighty-five percent clusters with only one single SSU rRNA sequence were removed from the dataset. The two-step clustering allowed 97% OTUs to be linked with 85% clusters, which were used for tree building.

### Comparison of metagenomic SSU rRNA to sequences in amplicon sequence databases

To facilitate detection of metagenomic SSU rRNA sequences matching sequences in the SILVA database (release 126) [12], all bacterial SSU rRNA sequences ≥ 1200 bp deposited in the SILVA database (release 126) [12] were added to the initial data set and a second clustering at 97% and consecutively 85% sequence similarity threshold was performed (version 9.1.13, -sort length -cluster_fast). Representative SSU rRNA sequences of 97% OTUs from the first clustering (without SILVA SSU rRNA sequences) were then matched to the 97% OTUs from the second clustering (with SILVA SSU rRNA sequences).

Considering the high level of contamination by chimeric sequences in the SILVA database [12], SILVA sequences were not counted towards cluster sizes, and clusters consisting only of SILVA sequences were excluded from further analysis (37,066 97% OTUs). The higher level of potentially chimeric sequences in the SILVA database was confirmed in our own chimera assessment with usearch (version 7.0, -uchime_ref) [19] using all high-quality SSU rRNA sequences extracted from genomes as training data (Additional file 3: Figure S2, data provided at https://bitbucket.org/berkeleylab/bacterialdiversity). Importantly, none of the representatives of 85% clusters exclusively comprising metagenomic SSU rRNA gene sequences that were used for tree construction were predicted to be chimeric (Additional file 3: Figure S2).

### Phylogenetic analysis

For tree building, 15 archaeal SSU rRNA gene sequences covering different archaeal phyla were added to the alignment as an outgroup. To identify SSU rRNA gene sequences of mitochondrial, plastid, archaeal, and eukaryotic origin, a phylogenetic tree was constructed with FastTree2 (version 2.1.9 SSE3, OpenMP) [20] and query sequences on long branches affiliated with Rickettsiales, Cyanobacteria, and Archaea or located between Bacteria and the archaeal outgroup were removed from the alignment. In the following, 1000 bootstrap replicate trees were generated with RAxML (version 8.2) [21] under the general time-reversible evolutionary model with gamma-distributed rates and a proportion of invariant sites (GTR+GAMMA+I). These bootstrap replicate trees were then used in RAxML (version 8.2, rogue_mr and MR_DROP options) [21] to identify and remove any 85% cluster whose position varied in a set of trees (“rogue taxa”) [22]. This procedure was repeated until less than five rogue taxa were left in the alignment. A final phylogenetic tree was constructed with RAxML (version 8.2) [21] GTR+GAMMA+I and 1000 bootstrap replicate trees were generated.

Cluster representatives were assigned to known bacterial phyla based on their position in the phylogenetic tree and branch support, taking into account existing taxonomic assignments of SSU rRNA sequence extracted from bacterial isolate genomes, SAGs, and MAGs in IMG/M [10]. Novel metagenomic SSU rRNA gene sequences were assigned to these phyla when they branched together with known references in a monophyletic clade.

### Phylogenetic diversity

For each of the major bacterial phyla, all 97% OTUs were extracted and two phylogenetic trees were constructed with RAxML (version 8.2) [21] under the general time-reversible evolutionary model with the CAT approximation of rate heterogeneity and a proportion of invariant sites (GTR+CAT+I). The first tree comprised all 97% OTUs in the respective phylum which were not exclusively found in metagenomic data, and the second tree was build based on all 97% OTUs. The sums of all branch lengths of both trees were calculated and compared to determine the increase in phylogenetic diversity (PD (Table S2).

### Primer mismatch analysis

All sequences from each 85% cluster were searched for mismatches to the current Earth Microbiome Project primer set (515f/806r) [14]. All sequences within each 85% cluster were treated as a group of sequences and passed as input into primer prospector [23]. The mean (overall weighted mismatch score) and standard deviation of scores per 85% cluster were calculated. Finally, if either forward or reverse primer had an overall weighted mismatch score > 1 [4], the sequence was predicted to be missed by the primer set.

## Additional files

Additional file 1: Table S1. Environmental origin of metagenomic datasets. Shown is the total number of metagenomes for each environment type, the total number of extracted SSU rRNA sequences > 1200 bp before and after quality filtering, and the total count of available basepairs in gigabases (Gb). (XLS 9 kb)

Additional file 2: Figure S1. Proportion of 97% OTUs and 85% clusters consisting of potentially chimeric SSU rRNA sequences exclusively found in metagenomes or in the SILVA database. (PNG 394 kb)

Additional file 3: Figure S2. Environmental reservoirs of newly detected bacterial lineages. Heatmaps show environmental distribution of bacterial phyla with lower taxonomic richness (> 30 and < 100 97% OTUs, upper panel) and higher taxonomic richness (> 100 97% OTUs, lower panel). Hierarchical clustering was used to group phyla and environments based on co-occurrence patterns. (PNG 87 kb)

Additional file 4: Table S2. Contribution of novel metagenomic lineages to taxonomic richness (TR) and phylogenetic diversity (PD) for major bacterial phyla. TR and PD are shown for phyla which contained at least 5 97% OTUs not taking into account metagenome-only 97% OTUs. (XLS 14 kb)

## Abbreviations

CPR: Candidate Phyla Radiation
GTR: General time-reversible model
I: Invariant sites
IMG/M: Integrated Microbial Genomes with Microbiome Samples
OTU: Operational taxonomic unit
PD: Phylogenetic diversity
SAG: Single amplified genome
SSU rRNA: Small subunit ribosomal ribonucleic acid
TR: Taxonomic richness

## References

[1] Lane DJ, Pace B, Olsen GJ, Stahl DA, Sogin ML, Pace NR (1985). Rapid determination of 16S ribosomal RNA sequences for phylogenetic analyses. Proc Natl Acad Sci U S A.

[2] Schloss PD, Girard RA, Martin T, Edwards J, Thrash JC (2016). Status of the archaeal and bacterial census: an update. MBio.

[3] Haas BJ, Gevers D, Earl AM, Feldgarden M, Ward DV, Giannoukos G (2011). Chimeric 16S rRNA sequence formation and detection in Sanger and 454-pyrosequenced PCR amplicons. Genome Res.

[4] Eloe-Fadrosh EA, Ivanova NN, Woyke T, Kyrpides NC (2016). Metagenomics uncovers gaps in amplicon-based detection of microbial diversity. Nat Microbiol.

[5] Wrighton KC, Thomas BC, Sharon I, Miller CS, Castelle CJ, VerBerkmoes NC (2012). Fermentation, hydrogen, and sulfur metabolism in multiple uncultivated bacterial phyla. Science.

[6] Rinke C, Schwientek P, Sczyrba A, Ivanova NN, Anderson IJ, Cheng J-F (2013). Insights into the phylogeny and coding potential of microbial dark matter. Nature.

[7] Brown CT, Hug LA, Thomas BC, Sharon I, Castelle CJ, Singh A (2015). Unusual biology across a group comprising more than 15% of domain bacteria. Nature.

[8] Hug LA, Baker BJ, Anantharaman K, Brown CT, Probst AJ, Castelle CJ (2016). A new view of the tree of life. Nat Microbiol.

[9] Anantharaman K, Brown CT, Hug LA, Sharon I, Castelle CJ, Probst AJ (2016). Thousands of microbial genomes shed light on interconnected biogeochemical processes in an aquifer system. Nat Commun.

[10] Chen I-MA, Markowitz VM, Chu K, Palaniappan K, Szeto E, Pillay M, et al. IMG/M: integrated genome and metagenome comparative data analysis system. Nucleic Acids Res. 2017;45:D507-16. 10.1093/nar/gkw929 PMC5210632 27738135

[11] Yarza P, Yilmaz P, Pruesse E, Glöckner FO, Ludwig W, Schleifer K-H (2014). Uniting the classification of cultured and uncultured bacteria and archaea using 16S rRNA gene sequences. Nat Rev Microbiol.

[12] Quast C, Pruesse E, Yilmaz P, Gerken J, Schweer T, Yarza P (2013). The SILVA ribosomal RNA gene database project: improved data processing and web-based tools. Nucleic Acids Res.

[13] Lang JM, Darling AE, Eisen JA (2013). Phylogeny of bacterial and archaeal genomes using conserved genes: supertrees and supermatrices. PLoS One.

[14] Caporaso JG, Lauber CL, Walters WA, Berg-Lyons D, Lozupone CA, Turnbaugh PJ (2011). Global patterns of 16S rRNA diversity at a depth of millions of sequences per sample. Proc Natl Acad Sci U S A.

[15] Mukherjee S, Stamatis D, Bertsch J, Ovchinnikova G, Verezemska O, Isbandi M (2017). Genomes OnLine Database (GOLD) v.6: data updates and feature enhancements. Nucleic Acids Res.

[16] Lagkouvardos I, Weinmaier T, Lauro FM, Cavicchioli R, Rattei T, Horn M. Integrating metagenomic and amplicon databases to resolve the phylogenetic and ecological diversity of the Chlamydiae. ISME J. 2014;8:115-25. 10.1038/ismej.2013.142 PMC3869019 23949660

[17] Nawrocki EP, Eddy SR (2013). Infernal 1.1: 100-fold faster RNA homology searches. Bioinformatics.

[18] Nawrocki EP, Burge SW, Bateman A, Daub J, Eberhardt RY, Eddy SR (2015). Rfam 12.0: updates to the RNA families database. Nucleic Acids Res.

[19] Edgar RC (2010). Search and clustering orders of magnitude faster than BLAST. Bioinforma Oxf Engl.

[20] Price MN, Dehal PS, Arkin AP. FastTree 2--approximately maximum-likelihood trees for large alignments. PLoS One. 2010;5:–e9490. 10.1371/journal.pone.0009490 PMC2835736 20224823

[21] Stamatakis A (2014). RAxML version 8: a tool for phylogenetic analysis and post-analysis of large phylogenies. Bioinformatics.

[22] Aberer AJ, Stamatakis A (2011). A simple and accurate method for rogue taxon identification. IEEE Int Conf Bioinforma Biomed.

[23] Walters WA, Caporaso JG, Lauber CL, Berg-Lyons D, Fierer N, Knight R (2011). PrimerProspector: de novo design and taxonomic analysis of barcoded polymerase chain reaction primers. Bioinformatics.

