# Correction to: Towards a balanced view of the bacterial tree of life

**DOI:** 10.1186/s40168-017-0367-2

**Published:** 2017-11-15

**Authors:** Frederik Schulz, Emiley A. Eloe-Fadrosh, Robert M. Bowers, Jessica Jarett, Torben Nielsen, Natalia N. Ivanova, Nikos C. Kyrpides, Tanja Woyke

**Affiliations:** Department of Energy Joint Genome Institute, Walnut Creek, CA USA

## Correction

Following publication of the original article [1], the authors pointed out that the figure shown as figure S1 is actually figure S2 and vice versa. Figure S1 should show the barcharts, and figure S2 should shows the heatmaps. Figure captions are in the correct order.

1. *“...SILVA-only OTUs (37,066 97% OTUs and 1266 85% clusters) (Additional file 3:* ***Figure S2*** *)...”* ***- >*** “*...SILVA-only OTUs (37,066 97% OTUs and 1266 85% clusters) (Additional file 3:* ***Figure S1*** *)...”*
2. *“...in groundwater and soil (Fig. 2, Additional file 2:* ***Figure S1*** *).”* ***->*** *“in groundwater and soil (Fig. 2, Additional file 2:* ***Figure S2*** *).”*
3. *“...from genomes as training data (Additional file 3:* ***Figure S2*** *, ...”* ***->*** *“...from genomes as training data (Additional file 3:* ***Figure S1*** *, ...”*
4. *“...were predicted to be chimeric (Additional file 3:* ***Figure S2*** *)” -> “...were predicted to be chimeric (Additional file 3:* ***Figure S1*** *)”*
