# Supplementary figures and images for: Towards a balanced view of the bacterial tree of life
Source: Microbiome. 2017 Oct 17;5:140. doi: 10.1186/s40168-017-0360-9 (PMC5644168; doi:10.1186/s40168-017-0360-9)

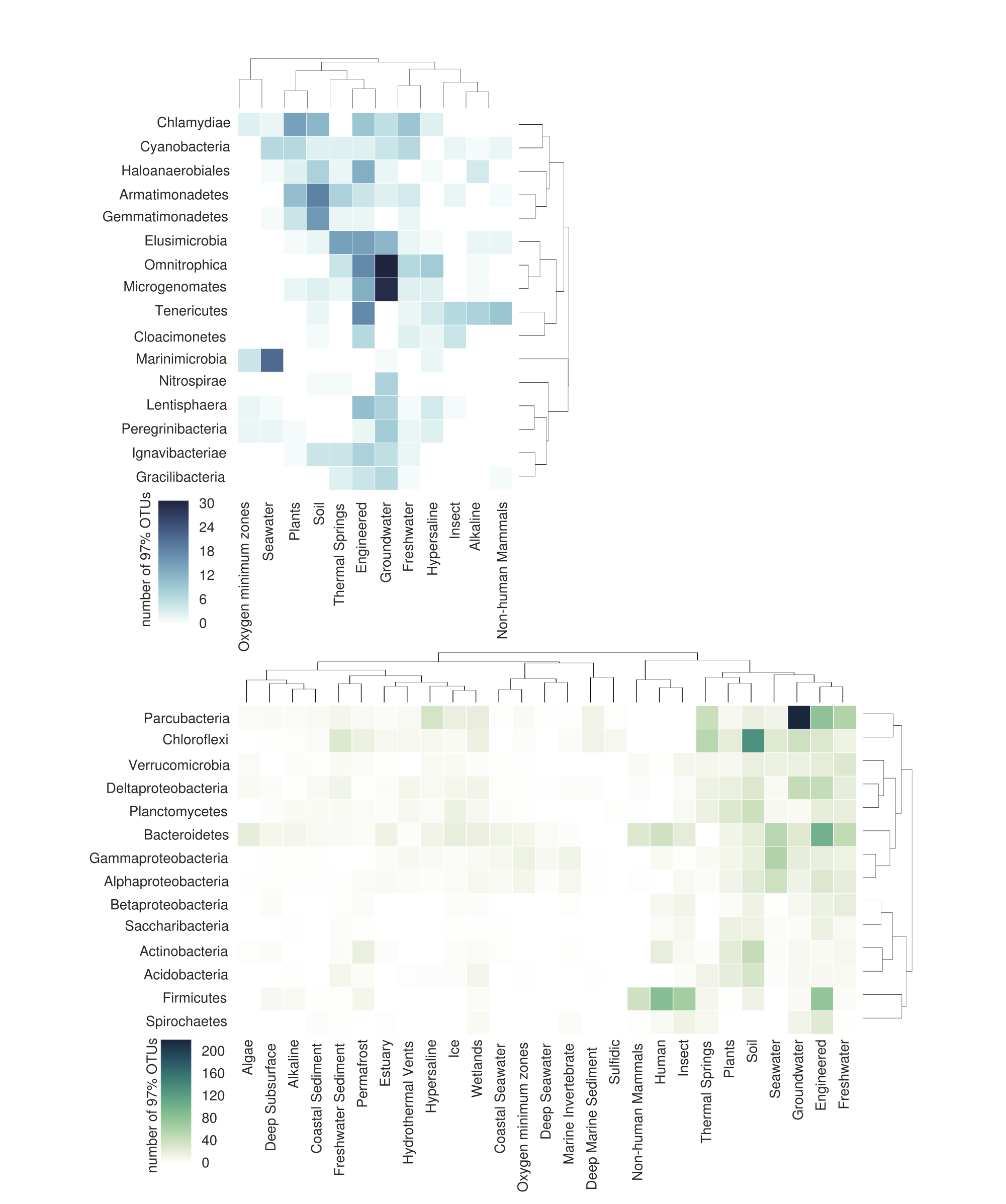

Supplement: Supplementary file 2 — Proportion of 97% OTUs and 85% clusters consisting of potentially chimeric SSU rRNA sequences exclusively found in metagenomes or in the SILVA database. (PNG 394 kb) [file 40168_2017_360_MOESM2_ESM.png]

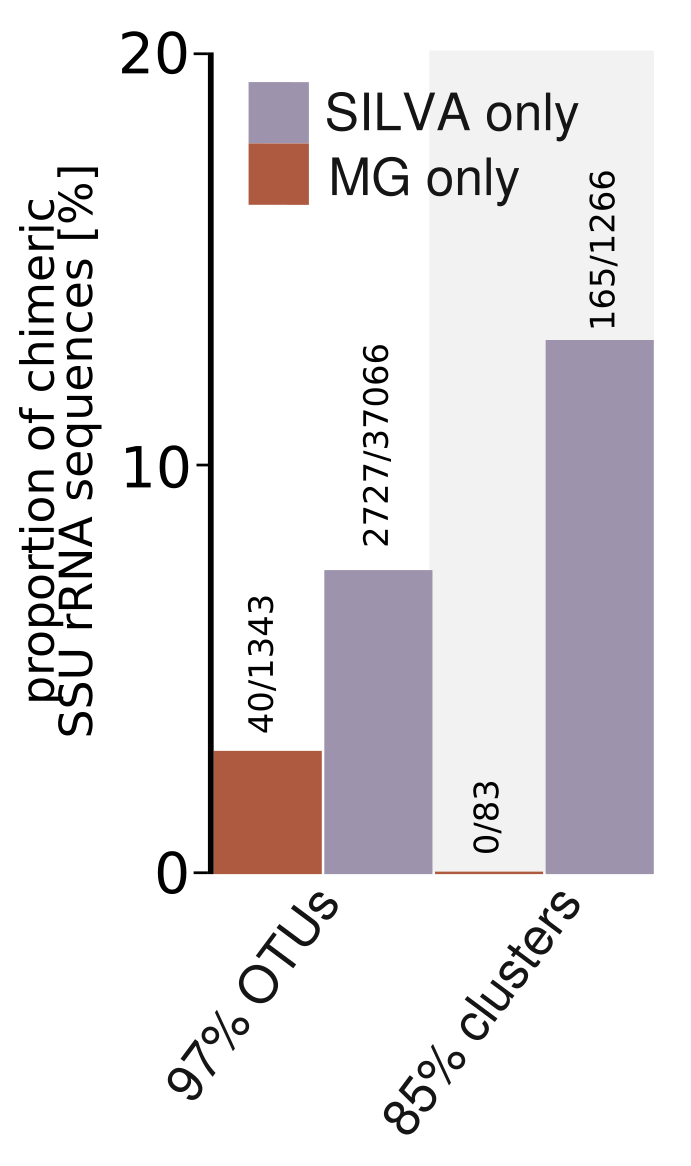

Supplement: Supplementary file 3 — Environmental reservoirs of newly detected bacterial lineages. Heatmaps show environmental distribution of bacterial phyla with lower taxonomic richness (> 30 and < 100 97% OTUs, upper panel) and higher taxonomic richness (> 100 97% OTUs, lower panel). Hierarchical clustering was used to group phyla and environments based on co-occurrence patterns. (PNG 87 kb) [file 40168_2017_360_MOESM3_ESM.png]
